# Overexpression of hypoxia-inducible factor-1α in hidradenitis suppurativa: the link between deviated immunity and metabolism

**DOI:** 10.1007/s00403-023-02594-6

**Published:** 2023-03-24

**Authors:** Naglaa Fathi Agamia, Osama Ahmed Sorror, Naglaa Mohamed Sayed, Rasha Abdelmawla Ghazala, Sammar Mohamed Echy, Doaa Helmy Moussa, Bodo Clemens Melnik

**Affiliations:** 1 Department of Dermatology, Andrology and Venereology, Faculty of Medicine, University of Alexandria, Alexandria, Egypt; 2 Department of Medical Biochemistry, Faculty of Medicine, University of Alexandria, Alexandria, Egypt; 3 Department of Clinical Pathology, Faculty of Medicine, University of Alexandria, Alexandria, Egypt; 4 Department of Dermatology, Environmental Medicine and Health Theory, University of Osnabrück, Osnabrück, Germany

**Keywords:** Glycolysis, Hidradenitis suppurativa, Hypoxia-inducible factor 1α, Keratinocyte proliferation, Th17 cells

## Abstract

Hypoxia-inducible factor-1α (HIF-1α) is the master transcription factor of glycolysis, Th17 cell differentiation and suppression of regulatory T cells. In the skin and serum of patients with psoriasis vulgaris, increased expression of HIF-1α has been reported, whereas HIF-1α expression in the skin and serum of patients with hidradenitis suppurativa (HS) has not yet been studied. The objective of the study is to demonstrate is there a role for HIF-1α in the pathogenesis of hidradenitis suppurativa, and its relation to HS severity. Twenty patients suffering from hidradenitis suppurativa were included in the study. Punch biopsies were taken from lesional skin for the determination of HIF-1α expression by immunohistochemical staining, and HIF-1α gene expression by quantitative reverse transcription real time PCR. Quantification of HIF-1α protein concentration was done by enzyme-linked immunosorbent assay. Twenty socio-demographically cross-matched healthy volunteers served as controls. We found increased serum levels of HIF-1α. Literature-derived evidence indicates that the major clinical triggering factors of HS, obesity, and smoking are associated with hypoxia and enhanced HIF-1α expression. Pro-inflammatory cytokines such as tumor necrosis factor-$a$ via upregulation of nuclear factor $\kappa$B enhance HIF-1α expression. HIF-1α plays an important role for keratinocyte proliferation, especially for keratinocytes of the anagen hair follicle, which requires abundant glycolysis providing sufficient precursors molecules for biosynthetic pathways. Metformin via inhibition of mTORC1 as well as adalimumab attenuate HIF-1α expression, the key mediator between Th17-driven deviated immunity and keratinocyte hyperproliferation. In accordance with psoriasis, our study identifies HS as an HIF-1α-driven inflammatory skin disease and offers a new rationale for the prevention and treatment of HS by targeting HIF-1$a$ overexpression.

## Introduction

Hidradenitis suppurativa (HS) is a chronic disabling inflammatory skin disease characterized by painful, deeply seated nodules, abscesses, sinuses, and scars with yet uncertain etiopathogenesis [1, 2]. The majority of HS patients are sporadic cases, whereas familial HS has accounted for 3.2–35.8% of HS patients, respectively [3, 4]. Clinical experience indicates that HS is triggered by environmental insults in genetically predisposed individuals. Obesity and cigarette smoking are among the most important triggering factors [5]. Increased activity of mechanistic target of rapamycin complex 1 (mTORC1) has been observed in the skin of HS [6], psoriatic epidermis [7, 8], obesity and diabetes mellitus [9, 10], and is regarded as a potential link between deviations of metabolism and immunity in HS [11–13]. Notably, hypoxia-inducible factor-1$\mathrm{a}$ (HIF-1$\mathrm{a}$) is a downstream effector of mTORC1 [14]. Overactivation of mTORC1 drives Th17 cell-induced expression of interleukin 17 (IL-17) [15, 16]. The IL-17 pathway plays a key role in the pathogenesis of HS and psoriasis [17–21]. HS is characterized by dysregulation of Th17 and regulatory T (Treg) cells [21], also observed in other autoimmune comorbidities of HS [19]. Notably, HIF-1α directly promotes Th17 development through transcriptional activation of retinoic acid-related orphan receptor γt (RORγt), a key transcription factor that drives the differentiation of Th17 cells [22, 23]. In contrast, HIF-1α restricts the differentiation and function of Treg cells through binding to FoxP3 targeting it for degradation [22, 23]. HIF-1α plays a pivotal role in metabolic reprogramming in inflammation [24] and controls the activation of macrophages, neutrophils and dendritic cells, creating a pro-inflammatory microenvironment within autoinflammatory lesions [25].

HIF-1α is the master transcription factor of hypoxia and glycolysis [26, 27]. Glycolysis is the preferred source of energy and biosynthetic precursor availability for highly proliferating cells including Th17 cells [28], psoriatic keratinocytes [29, 30] and anagen hair follicle cells [31–33]. Perilesional skin of HS shows mild psoriasiform hyperplasia [34]. Excessive proliferation of outer root sheath keratinocytes has been observed in HS [35, 36].

Upregulated expression of HIF-1α has been detected in the skin and serum of patients with psoriasis [37, 38] and other Th17-mediated inflammatory diseases [25]. In accordance with HS, obesity and smoking are aggravating factors promoting psoriasis [39, 40]. Therefore, we wondered whether HIF-1α is also overexpressed in the skin and serum of patients with HS and whether HIF-1α may link obesity and smoking to Th17 cell-driven dysregulations of immunity and infundibular keratinocyte hyperproliferation.

## Materials and methods

### Patients

This study included 20 patients suffering from hidradenitis suppurativa and 20 socio-demographically cross-matched healthy controls. All participants were recruited from the Dermatology Outpatient Clinic of the Alexandria Main University Hospital. Approval by ethical committee as well as written informed consent was obtained from all patients and controls. All procedures were in accordance with the ethical standards of the institutional and/or national research committee and the 1964 Declaration of Helsinki and registered with IRB No.: 00012098, FWA No.: 00018699. Patients with other concomitant lesions in the diseased area, patients who were receiving therapy for HS during the last 6 months, pregnant and lactating females were excluded. Patients were subjected to a full history, general medical and dermatological examination. Severity of HS was graded by the Hurley system: stage I: solitary or multiple, isolated abscess formation without scarring or sinus tracts; stage II: recurrent abscesses, single or multiple widely separated lesions, with sinus tract formation; stage III: diffuse or broad involvement, with multiple interconnected sinus tracts and abscesses [41].

### Skin biopsy

The procedure was explained to all patients. One 5 mm punch biopsy (for the immunohisto-chemical study) and two 2.5 mm punch biopsies (for ELISA and PCR) were taken from lesional skin of the patients. Three 5 mm punch biopsies of normal skin were taken from control subjects who were undergoing surgical procedure in the groin region recruited from the plastic surgery department.

### Histopathology and immunohistochemistry

All specimens were prepared for immunohistochemical staining using mouse anti-human monoclonal HIF-1α antibody [42]. The immunohistochemical staining was performed using the labeled streptavidin–biotin complex method. Primary antibody: HIF-1α-antibody (Affinity biosciences cat # AF1009), streptavidin–HRP conjugate (Epredia™ UltraVision Quanto Detection HRP DAB–Cat# TL-060-QHD) was prepared according to the manufacturer’s instructions, DAB working solution was prepared from the submitted DAB stock solution (Epredia™ UltraVision Quanto Detection HRP DAB–Cat# TL-060-QHD) in a 1 mg/ml concentration. HIF-1α positivity was considered when both nuclear and cytoplasmic staining were identified. Computed image analysis using Leica Application Suite 4.12.0 (Leica Microsystems CMS, GmbH) for semi-quantification of the number of positively stained inflammatory cells in the entire tissue biopsy in relation to the total number of inflammatory cells was calculated and expressed as a percentage. The overall staining intensities with HIF-1α monoclonal antibodies were scored using digital image analysis with a computer-assisted light microscope. The image of each slide was captured using a 400 × objective lens. Images were viewed and recorded using an Olympus microscope (Olympus, Centre Valley, PA, U.S.A.) equipped with a spot digital camera (Spot Imaging Solutions, Sterling Heights, MI, U.S.A.) and MATLAB software (MathWorks, Natlick, MA, U.S.A.). The mean values of each reaction were based on the mean pixel number. The integrity of the color intensity was based on grey-level transition probabilities in digitized images from dark to light. The overall intensity of staining of slides stained with HIF-1α monoclonal antibody was scored according to nuclear or cytoplasmic expression into 0 if staining intensity was < 10%, + 1 if staining intensity was 10% ≤ 30%, + 2 if 31% ≤ 50% and + 3 if > 50% staining intensity [37].

### Enzyme-linked immunosorbent assay

For serum preparation, the whole blood was collected and allowed to clot by leaving it undisturbed at room temperature. This took 10–20 min. The clot removed by centrifuging at 2000–3000 rpm for 20 min. Skin biopsies were preserved at − 80 °C. After determination of sample weight and addition of PBS, pH 7.4, samples were homogenized by hand or grinders and finally centrifuged for 3 min at a speed of 10,000 r.p.m. to remove the supernatant. The ELISA kit (Abcam, ab171571) was for the determination of HIF-1α protein concentrations in serum and tissue. Antibodies labelled with enzyme were added for an incubation time of 60 min at 37 °C. After washing the plates and addition of Chromogen solution A, B, optical density (OD) values were measured for calculation of HIF-1α protein concentrations of the samples [37].

### Quantitative reverse transcription real-time PCR

Total RNA was extracted from 10 mg skin tissue after lysis and homogenization, using silicate gel technique provided by the RNeasy Mini Kit (Qiagen) [43]. The concentration and purity of RNA were measured at 260, 280 and 230 nm using Nano Drop 2000c spectrophotometer (Thermo Scientific, USA). A ratio of A260/A280 = 1.8–2.1 and A260/A230 = 1.8–2.1 indicates highly pure RNA. Total RNA was reverse transcribed into cDNA using high-capacity reverse transcriptase kit (Applied Biosystems™, USA, catalog no. 4368814). To detect HIF-1$\mathrm{\alpha }$ gene expression in tissue samples, primers had been matched to the mRNA sequences of the target genes (NCBI Blast software). GADPH was used as housekeeping gene [44]. The PCR amplification was performed in a 25 µl reaction volume including SYBR green PCR Master Mix (Applied Biosystems) using ABI 7900 sequence detector (Applied Biosystems). The reaction was performed with 10 min of initial stage to activate the DNA polymerase, followed by 40 cycles at 95 °C for 15 s and 60 °C for 1 min. Single product formation was confirmed by melting point analysis and comparative CT method was used to calculate relative gene expression with GADPH as an endogenous control. For statistical analysis of the CT values, 2^−ΔΔCT^ method was applied for each specific primer and real-time PCR [45].

## Results

### Patient data

The group of HS patients included 15 males and 5 females. Their mean age was 26.10 ± 6.10 years while the controls included 14 males and 6 females. Their mean age was 25.65 ± 4.59 years. There was no significant difference regarding sex and age. The mean duration of the disease was 12.0 ± 9.86 months. Patients had significantly higher BMI compared to controls. The mean BMI in the HS group was 29.49 ± 4.56 kg/m^2^, while BMI in the control group was 26.74 ± 3.10 kg/m^2^ (Table 1). With regard to Hurley stage, 25% (5 patients) were of stage I, 45% (9 patients) of stage II and 30% (6 patients) of stage III. HS clinical staging was found to have a significant relation to the duration of HS and BMI of the patients but no significant relation to sex, age, or smoking (Table 2).

**Table 1:** Comparison between HS patients and controls according to grading of the stain intensity and HIF-1α expression

	Patients (*n* = 20)	Control (*n* = 20)	Test of sig.	*p*
Grading of the stain intensity				
0	0 (0%)	4 (20%)	*χ*^2^ = 20.554*	^MC^*p* < 0.001*
+ 1	7 (35%)	16 (80%)		
+ 2	7 (35%)	0 (0%)		
+ 3	6 (30%)	0 (0%)		
HIF-1α serum protein concentration (pg/ml)				
Mean ± SD	5149.1 ± 587.6	2580.4 ± 562.8	*t* = 14.118*	< 0.001*
Median (Min.–Max.)	4992.8 (4267–6124)	2454.5 (1813–3685)		
HIF-1α tissue protein concentration (pg/ml)				
Mean ± SD	3205.4 ± 473.2	1727.3 ± 482.4	*t* = 9.782*	< 0.001*
Median (Min.–Max.)	3319 (2421–4074)	1809.5 (703.5–2322)		
*HIF1A* gene expression				
Mean ± SD	0.25 ± 0.16	0.85 ± 0.09	*t* = 14.698*	< 0.001*
Median (Min.–Max.)	0.23 (0.03–0.52)	0.85 (0.70–0.98)		

*p*: *p* value for comparing between the studied groups

*SD* standard deviation, *t* Student’s *t* test, χ^2^ Chi-square test, *MC* Monte Carlo

*Statistically significant at *p* ≤ 0.05

**Table 2:** Relation between HS stage and different parameters in patients’ group (*n* = 20)

	HS stage			Test of sig.	*p*
	Stage I (*n* = 5)	Stage II (*n* = 9)	Stage III (*n* = 6)		
Sex					
Male	4 (80%)	5 (55.6%)	6 (100%)	*χ*^2^ = 3.476	^MC^*p* = 0.147
Female	1 (20%)	4 (44.4%)	0 (0%)		
Age (years)					
Mean ± SD	25.80 ± 6.06	26.33 ± 7.81	26.0 ± 3.79	*F* = 0.012	0.988
Median (Min.–Max.)	22.0 (21.0–35.0)	27.0 (13.0–38.0)	25.5 (20.0–31.0)		
Duration (month)					
Mean ± SD	2.20 ± 0.84	8.78 ± 4.38	25.0 ± 4.52	*H* = 16.424*	< 0.001*
Median (Min.–Max.)	2.0 (1.0–3.0)	7.0 (5.0–18.0)	24.0 (18.0–30.0)		
Smoking					
Non-smoker	1 (20%)	5 (55.6%)	0 (0%)	*χ*^2^ = 5.082	0.057
Smoker	4 (80%)	4 (44.4%)	6 (100%)		
BMI (kg/m^2^)					
Mean ± SD	24.08 ± 1.68	28.67 ± 1.44	35.23 ± 1.82	*F* = 66.950*	< 0.001*
Median (Min.–Max.)	23.2 (22.6–26.0)	28.2 (26.8–31.1)	35.35 (32.8–37.1)		
Grading of the stain intensity					
+ 1	5 (100%)	2 (22.2%)	0 (0%)	*χ*^2^ = 24.295*	^MC^*p* < 0.001*
+ 2	0 (0%)	7 (77.8%)	0 (0%)		
+ 3	0 (0%)	0 (0%)	6 (100%)		

*p*: *p* value for comparing between different stages

*SD* standard deviation, *F* F for one-way ANOVA test, *H* H for Kruskal–Wallis test, *χ*^*2*^ Chi-square test, *MC* Monte Carlo

*Statistically significant at *p* ≤ 0.05

### Immunohistochemical detection of HIF-1α in lesional HS skin

**Fig. 1:**
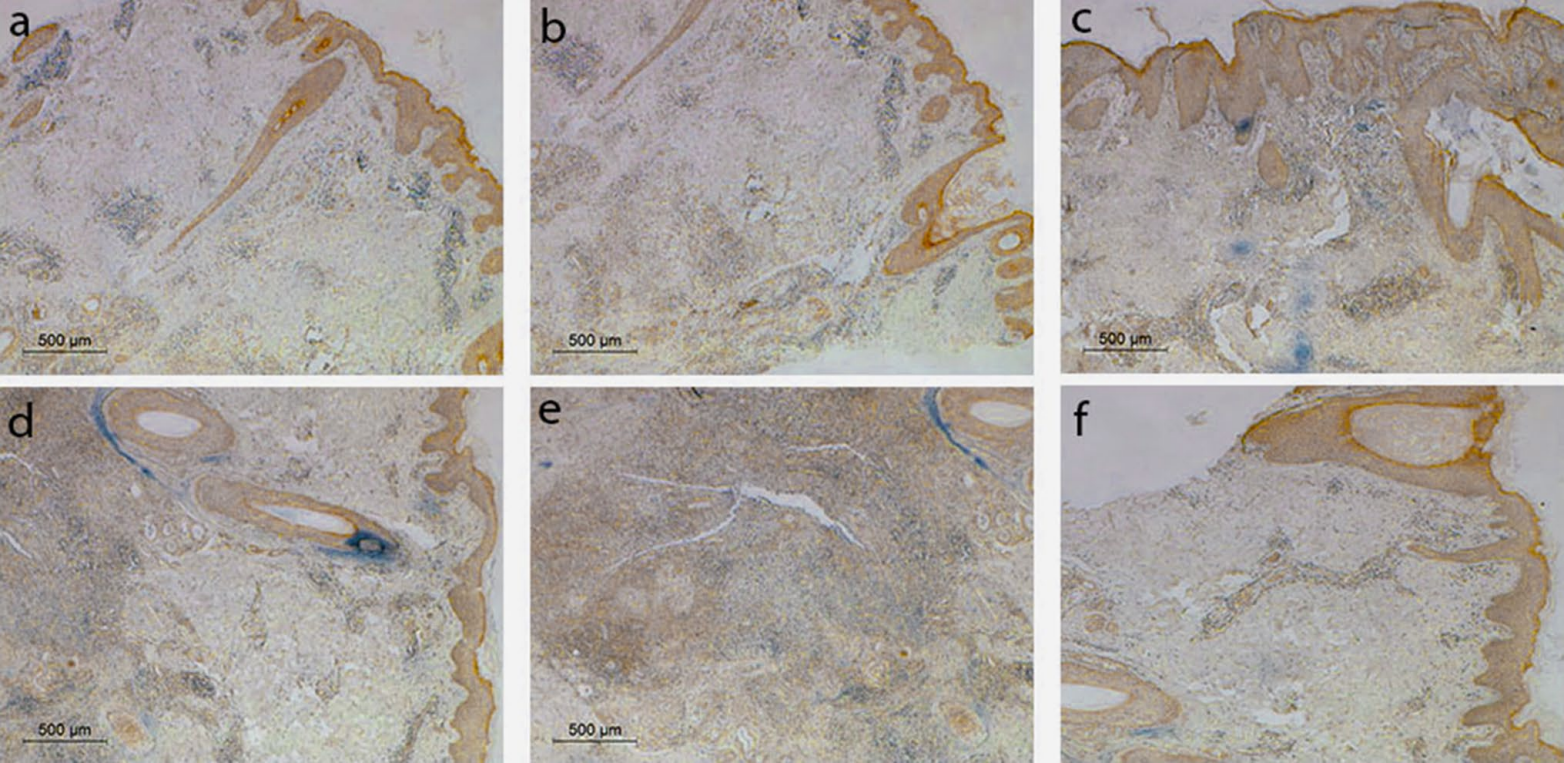
Immunohistochemical expression of HIF-1α in inflammatory cells in correlation to Hurley staging. **a-e** The grade of stain intensity in the inflammatory infiltrate is increased with the increase in Hurly staging. **f** Immunohistochemical expression of HIF-1α in controls. **a** HIF-1α expression in skin biopsy from patient with HS stage I of Hurley system showing grade 0 stain intensity of the inflammatory infiltrate (< 10%). **b** HIF-1α expression in skin biopsy from patient with HS stage I of Hurley system showing grade + 1 stain intensity of the inflammatory infiltrate (10% ≤ 30%). **c** HIF-1α expression in skin biopsy from patient with HS Hurley stage II exhibits grade + 2 stain intensity of the inflammatory infiltrate (31% ≤ 50%). **d** HIF-1α expression in skin biopsy from HS patient with Hurley stage III shows grade + 3 stain intensity of the inflammatory infiltrate (> 50%). **e** HIF-1α in HS Hurley stage III of shows grade + 3 stain intensity in the deep dermis

Stain intensity in the HS group (35% score + 1, 35% score + 2, 30% score + 3) was significantly higher compared to the control group (20% score 0; 80% score + 1) (Table 1). Figure 1 and Table 2 show the representative of immunohistochemical expression of HIF-1α in relation to Hurley staging (Fig. 1a–e). An increased HIF-1α immune staining of the inflammatory infiltrate could be observed in relation to Hurley stage, while Fig. 1f represents immunohistochemical expression of HIF-1α in controls.

### HIF-1α protein concentration in lesional HS skin

**Fig. 2:**
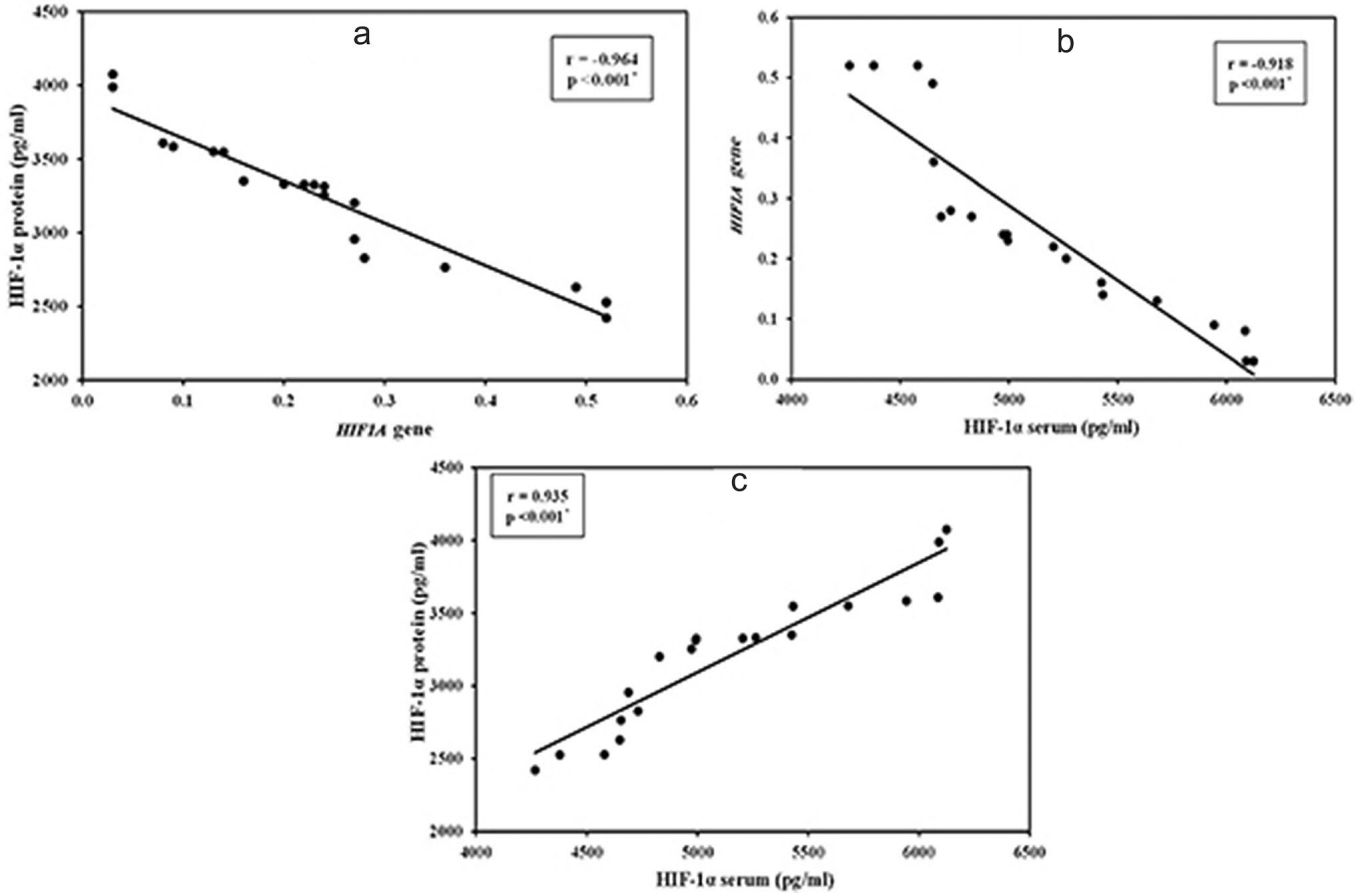
Correlations between HIF-1α skin protein, serum and gene expression. **a** Correlation between HIF-1α protein levels in HS skin and HIF-1α gene expression. **b** Correlation between HIF-1α gene vs. HIF-1α serum in patients’ group. **c** Correlation between HIF-1α protein vs. HIF-1α serum expression levels in patients’ group

The cutaneous HIF-1α protein in lesional skin of HS patients (3205.4 ± 473.2 pg/ml) was significantly increased compared to healthy controls (1727.3 ± 482.4 pg/ml) (*p* < 0.001) (Table 1). There was a statistically significant correlation between grading of the stain intensity (Table 3) and Hurley staging of HS (Table 4) and HIF-1α serum level (*p* < 0.001) (Fig. 2c).

**Table 3:** Relation between grading of the HIF-1α stain intensity with HIF-1α expression in patient’s group (*n* = 20)

HIF-1α expression	Grading of the stain intensity			*F*	*P*
	+ 1 (*n* = 7)	+ 2 (*n* = 7)	+ 3 (*n* = 6)		
HIF-1α serum protein concentration (pg/ml)					
Mean ± SD	4598.5 ± 228.3	5062.5 ± 247.2	5892.5 ± 279.2	43.650*	< 0.001*
Median (Min.–Max.)	4649 (4267–4973)	4994.5 (4731–5425)	6014.5 (5432–6124)		
HIF-1α tissue protein concentration(pg/ml)					
Mean ± SD	2725.6 ± 292.8	3239.4 ± 188.0	3725.3 ± 239.4	27.206*	< 0.001*
Median (Min.–Max.)	2629 (2421–3254)	3324 (2828–3351)	3596 (3547–4074)		
*HIF1A* gene expression					
Mean ± SD	0.42 ± 0.12	0.23 ± 0.04	0.08 ± 0.05	27.752*	< 0.001*
Median (Min.–Max.)	0.49 (0.24–0.52)	0.23 (0.16–0.28)	0.09 (0.03–0.14)		

*p*: *p* value for comparing between different grading

*SD* standard deviation, *F* F for one-way ANOVA test

*Statistically significant at *p* ≤ 0.05

**Table 4:** Relation between HS stage and HIF-1α expression in patients’ group (*n* = 20)

HIF-1α expression	HS stage			*F*	*p*
	Stage I (*n* = 5)	Stage II (*n* = 9)	Stage III (*n* = 6)		
HIF-1α serum protein concentration (pg/ml)					
Mean ± SD	4505.6 ± 174.2	5011.0 ± 247.6	5892.5 ± 279.2	47.163*	< 0.001*
Median (Min.–Max.)	4580 (4267–4654)	4991 (4688.5–5425)	6014.5 (5432–6124)		
HIF-1α tissue protein concentration (pg/ml)					
Mean ± SD	2573.8 ± 129.29	3209.6 ± 188.6	3725.3 ± 239.4	48.197*	< 0.001*
Median (Min.–Max.)	2529 (2421–2764)	3314 (2828–3351)	3596 (3547–4074)		
*HIF1A* gene expression					
Mean ± SD	0.48 ± 0.07	0.23 ± 0.04	0.08 ± 0.05	89.706*	< 0.001*
Median (Min.–Max.)	0.52 (0.36–0.52)	0.24 (0.16–0.28)	0.09 (0.03–0.14)		

*p*: *p* value for comparing between different grading

*SD* standard deviation, *F* F for one-way ANOVA test

*Statistically significant at *p* ≤ 0.05

### Serum concentrations of HIF-1α

The mean serum HIF-1α levels in HS patients (5149.1 ± 587.6 pg/ml) was significantly increased compared to the control group (2580.4 ± 562.8 pg/ml) (*p* < 0.001) (Table 1). There was also a positive correlation between HIF-1α serum levels with Hurley staging of HS (Table 4) as well as HIF-1α protein expression (Fig. 2c) and immunohistochemical expression in skin biopsies (Table 3).

### Lesional HIF1α gene expression

Relative gene expression of HIF1A was lower in the HS group (0.25 ± 0.16) compared to controls (0.85 ± 0.09) (*p* < 0.001) (Table 1). Notably, HIF1A gene expression showed a negative correlation to both HIF-1α protein expression in the skin (*p* < 0.001) (Fig. 2a) and HIF-1α serum levels (*p* < 0.001) (Fig. 2b).

## Discussion

Our study is the first investigation showing increased expression of HIF-1$\mathrm{a}$ in lesional skin of HS patients. In normal human skin, HIF-1a protein expression is low and focal in the epidermis in contrast to hair follicles, sebaceous glands, and sweat glands, where HIF-1α is abundantly expressed [37]. Upregulated expressions of HIF-1α has been detected in psoriasis vulgaris [37, 38, 46–48] and other autoinflammatory diseases related to Th17-mediated inflammation [25, 49–51]. HIF-1α plays a pivotal role in Th17 cell differentiation [22, 23]. HS exhibits hyperproliferation of ORS keratinocytes [35, 36] and is associated with Th17-mediated autoimmunity [17–19, 52, 53].

HIF-1$\mathrm{a}$ is the key transcription factor of glycolysis [54, 55], which is required for accelerated cell proliferation [26]. HIF**-**1α-induced glycolysis has been associated with keratinocyte proliferation in psoriasis vulgaris [29, 30, 47]. Notably, the human hair follicle is intensively engaged in aerobic glycolysis [32, 33] and exhibits high expression of HIF-1$\mathrm{a}$ [37]. The pathogenic role of HIF-1$\mathrm{a}$ in HS is supported by our observation of increased expression of HIF-1$\mathrm{a}$ in lesional skin of HS associated with a positive correlation with Hurley staging (Table 2). In analogy to psoriasis [38], we found also significantly elevated serum levels of HIF-1$\mathrm{a}$ in our HS patients compared to healthy controls. In psoriasis, high serum levels of HIF-1α showed a correlation with overexpression of IL-6 [38]. IL-6 via STAT3 signaling enhances HIF-1$\mathrm{a}$ expression [22].

In psoriasis, human dermal microvascular endothelial cells display increased angiogenesis and migration [56]. In the dermis of lesional HS areas with chronic inflammation, increased neovascularization has also been observed [57, 58]. Enhanced vascular endothelial growth factor (VEGF) expression has been reported in psoriasis and HS [59]. HIF-1 is a master regulator of angiogenesis and participates in vasculature formation by synergistic correlations with other proangiogenic factors including VEGF [60].

Translational evidence indicates that overexpression of HIF-1 signaling is related to obesity and smoking, key clinical triggering factors of HS. Increased oxygen consumption of adipocytes in obesity has been shown to enhance HIF-1α expression [61]. In contrast to elevated HIF-1α protein levels in patients with HS, we observed reduced HIF-1α mRNA levels, an unexpected finding that, however, fits well to observations in human endothelial cells exposed to chronic hypoxia that progressively decreases HIF-1α mRNA while HIF-1α protein levels rapidly peak after hours and then slowly decay [62, 63]. Noteworthy, microRNA-21 (miR-21) is upregulated in adipose tissue of obese and diabetic subjects [64–66]. A significant overexpression of miR-21, miR-155, miR-223, miR-31, miR-125b, and miR-146a has been observed in lesional HS skin compared to healthy controls [67]. Intriguingly, miR-21 targets and thus attenuates the expression of VHL mRNA [68–71]. MiR-146a is upregulated by NF$\upkappa$B and targets 3´UTRs of signaling proteins of innate immune responses [72] as well as HIF-1α mRNA [73]. MiR-148a is another upregulated miR related to obesity and diabetes [74–78]. Notably, HIF1AN, the gene encoding FIH-1, is a direct target of miR-148a, miR-31 and miR-125 that all inhibit HIF-1$\mathrm{a}$ transactivation (TargetScanHuman, release 8.0).

Chronic cigarette (CS) smoke exposure induces systemic hypoxia [79] CS extract also increased the expression of miR-21 and HIF-1$\mathrm{a}$ in human bronchial epithelial (HBE) cells [80]. HBE cells release miR-21-enriched exosomes after CS exposure enhancing HIF-1α signaling via targeting pVHL [81, 82]. Further evidence confirms that CS activates HIF-1$\mathrm{a}$ [83, 84]. Nicotine increased HIF-1$\mathrm{a}$ expression in non-small cell lung cancer cells [85]. Benzo(a)pyrene, a component of CS extract [86], enhances the binding ability of HIF-1α to HIF-1β protein [87]. CS and hypoxia both increase oxidative stress and produce reactive oxygen species, which induce autoreactive pro-inflammatory T cells and reduce Treg cell activity [88].

Interestingly, vitamin D deficiency has been repeatedly confirmed in HS patients and has been related to disease severity [89–93]. Vitamin D has inhibitory effects on mTORC1 [94, 95] which promotes the synthesis of HIF-1$\mathrm{a}$ [14]. Vitamin D supplementation downregulated mTORC1 activity and lowered HIF-1$\mathrm{a}$ mRNA levels in CD4 + T cell subsets of high-fat-diet-induced obese mice [96]. Of note, vitamin D/VDR signaling enhances the transcription of VHL [97].

Pro-inflammatory cytokines, such as IL-17A, tumor necrosis factor-$\mathrm{a}$ (TNF-$\mathrm{a}$), and predominantly IL-1$\upbeta$ are markedly increased in HS lesional skin [98]. IL-1$\upbeta$ upregulates HIF-1$\mathrm{a}$ and HIF-1$\mathrm{a}$-dependent gene expression [99, 100]. Inhibition of IL-1 by anakinra showed therapeutic effects in severe HS [101]. In HepG2 cells, IL-1$\upbeta$ had no effect on reporter gene expression in normoxia, whereas during hypoxia IL-1$\upbeta$ amplified HIF-1 reporter gene activity by 25% compared with hypoxia alone [102]. HIF-1$\mathrm{a}$ has been identified as target gene of NF-$\upkappa$B linking hypoxia, inflammation and oxidative stress [103–106]. NF-$\mathrm{k}$B upregulated via TNF$\mathrm{a}$ directly enhances the expression of HIF-1$\upbeta$ mRNA and protein in an evolutionarily conserved manner [107]. It has recently been demonstrated in experimental autoimmune encephalomyelitis (EAE) that IL-17A recruits IL-1β-secreting myeloid cells that prime pathogenic γδT17 and Th17 cells [108], whereas mice with HIF-1α-deficient T cells are resistant to induction of Th17-dependent EAE [23]. These data underline an intimate crosstalk between pro-inflammatory cytokines and HIF-1 signaling, which may also have an impact on HS pathogenesis.

Single-cell RNA sequencing reveals cellular and transcriptional changes associated with M1 macrophage polarization in HS related to increased expression of HIF-1$\mathrm{a}$ [109]. HIF-1$\mathrm{a}$ plays a key role in the induction of macrophage glycolysis and activation of pro-inflammatory M1 polarization [110]. In M1 polarized macrophages, HIF-1$\mathrm{a}$ is responsible for sustanined production of IL-1$\upbeta$ [111].

Recent evidence indicates that glycolysis is coordinated by both Notch and HIF-1$\mathrm{a}$ signaling [112]. Notch intracellular domain (ICD) enhances recruitment of HIF-1 $\mathrm{a}$ to its target promoters [113]. HIF-1α stabilizes Notch signaling [114–116]. Overexpressed Notch/PI3K/AKT [3] and mTORC1 signaling in HS [6] may thus further enhance HIF-1-mediated gene regulation in HS.

Infundibular hyperkeratosis with subsequent follicular plugging in intertriginous skin areas may result in ductal hypoxia, an HIF-1$\mathrm{a}$-induced comedogenic mechanism earlier suggested in acne pathogenesis [117, 118]. In fact, hyperbaric oxygen treatment (HBOT) improves HS and enhances the efficacy of adalimumab and ustekinumab [119–121]. In selected experimental models, HBOT decreased the expression of HIF-1$\mathrm{a}$ [122–124].

There is recent interest in the antidiabetic drug metformin for the treatment of HS [125–130]. Metformin not only attenuates the activity of mTORC1 [131] but downregulates the expression of HIF-1$\mathrm{a}$ [132–137]. Inhibition of mTORC1 by rapamycin (sirolimus) as well improved the clinical course of HS [138].

**Fig. 3:**
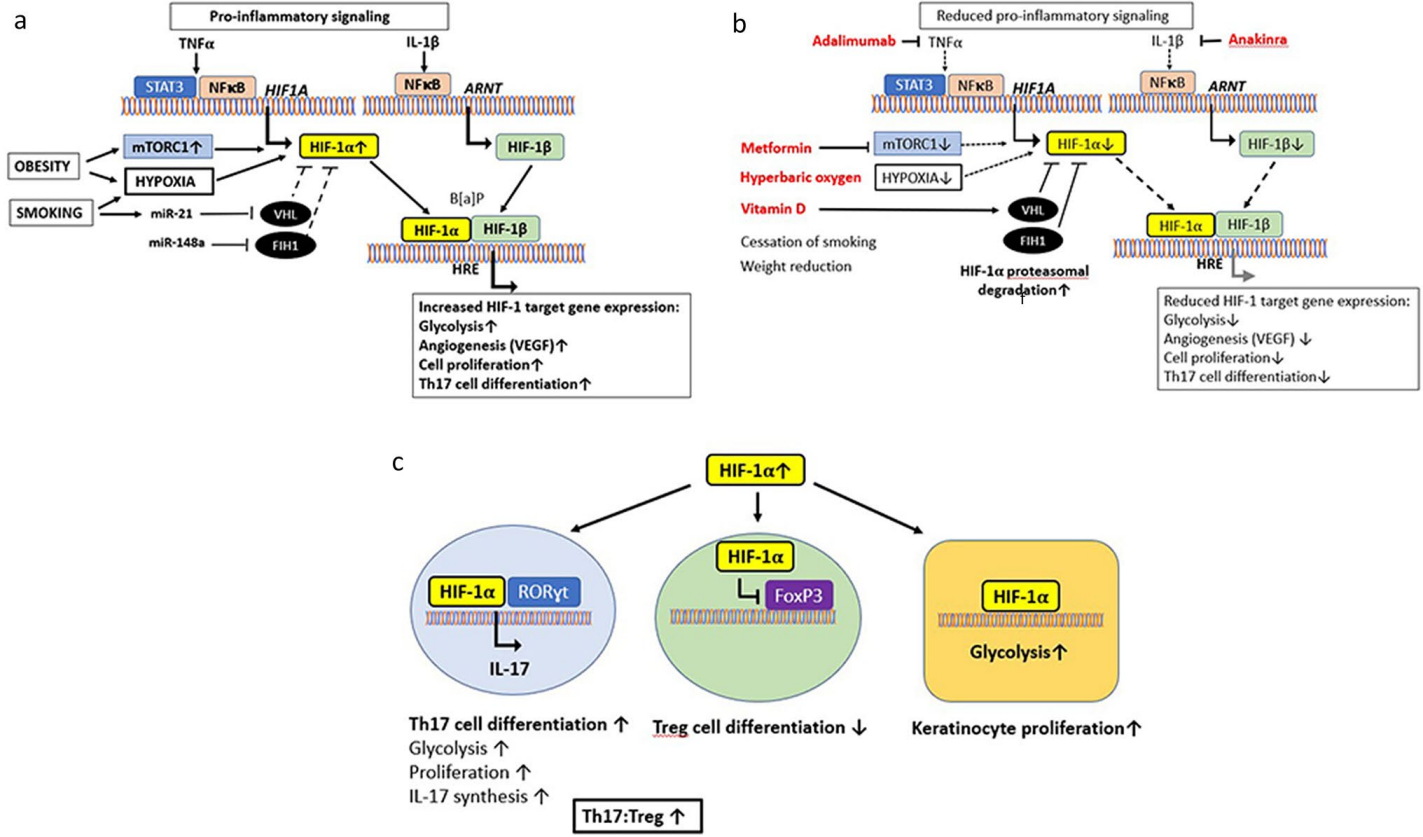
Illustrates HIF-1$a$ signaling in hidradenitis suppurativa (HS) and potential pharmacological targeting of HIF1-$\alpha$ in HS. **a** HIF-1-mediated gene expression is induced after formation of the heterodimer complex of HIF-1α and HIF-1$\beta$ (aryl hydrocarbon receptor nuclear translocator ARNT). The HIF-1α/HIF-1$\beta$ complex binds to hypoxia response elements (HREs) to regulate gene expression (Gunton, 2020; Ke and Costa, 2006; Ruas and Poellinger, 2005; Semenza et al*.*, 2006). HIF-1α plays a crucial role in oxygen sensing (Fandrey et al*.*, 2006; Huang et al*.*, 1996; Ratcliffe et al*.*, 1998; Schofield and Ratcliffe, 2004; Zagórska and Dulak, 2004). In the presence of oxygen, HIF-1α is hydroxylated by prolyl hydroxylase domain proteins, which function as oxygen sensors to regulate HIF degradation mediated by von Hippel-Lindau (VHL) protein that targets HIF-1α to ubiquitination (Ruas and Poellinger, 2005; Semenza et al*.*, 2006; Yuan et al*.*, 2003). Factor inhibiting HIF-1 (FIH-1) inhibits HIF-1α transactivation (Mahon et al*.*, 2001; Wang et al*.*, 2014). Obesity induces hypoxia and mTORC1 increasing HIF-1α expression. **b** Adalimumab and anakinra attenuate NF$k$B-mediated HIF-1 signaling. Metformin via suppression of mTORC1 attenuates HIF-1$a$ translation. Hyperbaric oxygen reduces hypoxia. Vitamin D enhances the expression of VHL, which promotes HIF-1$a$ degradation. **c** HIF-1$a$ activates the expression of retinoic acid-related orphan receptor γt (RORγt), which promotes Ht17 cell differentiation and inhibits FoxP3 attenuating the activity of regulatory T cells (Treg). HIF-1$a$-stimulated glycolysis enhances keratinocyte proliferation

Taken together, our study provides evidence for increased lesional HIF-1$\mathrm{a}$ protein expression in patients with HS that correlates with Hurley stage (Tables 2, 4). In accordance with the autoimmune pathogenesis of psoriasis [37], we observed increased HIF-1$\mathrm{a}$ protein expression in HS, which both share enhanced HIF-1$\mathrm{a}$ and IL-17 signaling (Fig. 3). There is compelling evidence that HIF-1$\mathrm{a}$ is a dysregulated master transcription factor of HS pathogenesis explaining (1) enhanced HIF-1$\mathrm{a}$-driven glycolysis with keratinocyte hyperproliferation, (2) increased HIF-1$\mathrm{a}$/ROR$\upgamma$t-mediated Th17 cell differentiation with increased IL-17 production, (3) reduced Treg cell differentiation by HIF-1$\mathrm{a}$-mediated degradation of FoxP3, (4) HS aggravation by obesity and smoking, key trigger factors of HS that increase HIF signaling. Apparently, lesional imbalances HIF-1 signaling are at the center of disturbed infundibular keratinocyte and Th17 cell proliferation in the pathogenesis of HS. Pharmacological targeting of HIF-1$\mathrm{a}$ may be a promising approach to manage HS as already suggested for psoriasis and other autoimmune disorders [48, 50, 139, 140].

## Data Availability

All data analyzed in this study are included in the published article as Dataset S1 and Dataset S2.
